# The Assisted Breathing before Cord Clamping (ABC) Study Protocol

**DOI:** 10.3390/children8050336

**Published:** 2021-04-26

**Authors:** Michael P. Meyer, Elizabeth Nevill

**Affiliations:** 1 Neonatal Unit, KidzFirst, Middlemore Hospital, Auckland 2025, New Zealand; elizabeth.nevill@cmdhb.org.nz; 2 Department of Paediatrics, University of Auckland, Auckland 2025, New Zealand

**Keywords:** preterm, delayed cord clamping, resuscitation, clinical trial, protocol, red blood cell transfusion, preterm cardiovascular transition

## Abstract

Major physiologic changes occur during the transition after birth. For preterm infants, current understanding favours allowing the initial changes to occur prior to cord clamping. Amongst other improved outcomes, systematic reviews have indicated a significant reduction in neonatal blood transfusions following delayed cord clamping. This may be due to a placental transfusion, facilitated by the onset of respiration. If breathing is compromised, placental transfusion may be reduced, resulting in a greater red cell transfusion rate. We designed a randomised trial to investigate whether assisting respiration in this high-risk group of babies would decrease blood transfusion and improve outcomes. The Assisted Breathing before Cord Clamping (ABC) study is a single-centre randomised controlled trial. Preterm infants < 31 weeks that have not established regular breathing before 15 s are randomised to a standard or intervention group. The intervention is intermittent positive pressure ventilation via T piece for 30 s, whilst standard management consists of 30 s of positioning and gentle stimulation. The cord is clamped at 50 s in both groups. The primary outcome is the proportion of infants in each group receiving blood transfusion during the neonatal admission. Secondary outcomes include requirement for resuscitation, the assessment of circulatory status and neonatal outcomes.

## 1. Introduction

Major physiologic changes occur during the transition immediately after birth. These include lung aeration and expansion, which is associated with a marked and rapid reduction in pulmonary vascular resistance and increased pulmonary blood flow [1]. This changes the direction of the ductal shunt so that it is primarily left to right, and the systemic blood flow is largely derived from the increased left ventricular output. The in utero vascular resistance is low because of the placental bed. Cord clamping has the potential to change this vascular resistance, particularly if the lungs have not been expanded [2,3]. Preterm infants, who may not rapidly establish adequate lung aeration before cord clamping, are at a high risk of perturbations to this normal process especially if the cord is clamped early. Indeed, clamping the preterm cord after a delay of at least 30 s has been shown in a number of systematic reviews to improve survival, although this finding was recently noted to be of borderline significance [4,5,6]. As noted above, however, the critical factor in the timing of the cord clamping may be related to the onset of respiration rather than the time elapsed. Whilst this was suggested years ago [7,8], there has been a recent resurgence of studies examining “physiologic” cord clamping once breathing is fully established [9,10]. To date, most of these studies are small feasibility studies, although larger clinical studies are underway or have been proposed [11]. If the cord is not clamped immediately (or “early”) and clamping is “deferred” or “delayed” (DCC) for a period of time (e.g., 30 to 60 s), many infants, including those that are very preterm, will be breathing before clamping. In a recent study of infants < 32 weeks, this amounted to approximately 70% at 30 s and 90% by 60 s [12,13]. On the other hand, if preterm infants are not breathing before clamping, they may be deemed to be in need of resuscitation, hence the term “neonatal resuscitation with an intact cord” [1].

The finding of some importance and consistency in meta-analysis in relation to DCC in preterm infants is a reduction in neonatal blood transfusion. The overall relative risk for DCC compared to early clamping was 0.81 (0.74–0.87) with moderate heterogeneity (61%). For infants < 28 weeks, the risk was 0.91 (0.85–0.97) with 39% heterogeneity [4]. The mean haematocrit increase in the DCC group was 2.7%. The largest study included in the meta-analysis contributed to over 50% of the cases and showed a significant reduction of 10% in the proportion of infants transfused [14]. The other relatively large study was the UK Cord Trial [15], where infants < 32 weeks were randomised to 2 min or more DCC compared to clamping before 20 s. Neonatal outcomes were assessed in 266 infants with a similar reduction in the proportion of transfused infants from 54 to 47%, although this difference was not significant. The number of infants transfused for early hypotension in this study was, however, significantly decreased from 6/132 to 0/134 (*p* = 0. 014).

Although the reasons for neonatal transfusion are multifactorial, there is evidence of a transfer of blood from the placenta (a placental transfusion) to the infant in cases where DCC occurs, and this may be one mechanism for the observed reduction in neonatal transfusions. Although a few studies have directly measured the infant’s blood volume, most of the evidence for placental transfusion is indirect [16,17]. There is an increase in haematocrit in many randomised trials (where the control group did not have DCC), an increase in body weight demonstrated by continuous weighing after birth with the cord intact and a corresponding decrease in placental weight compared to cases where no DCC occurred [4,16,18]. In addition, many earlier studies measured the residual placental blood volume (RPV) by allowing blood from the placenta to drain under gravity into a graduated measuring cylinder after delivery [19], DCC was associated with a reduction in the RPV. Important factors determining the RPV, i.e., the inferred amount of placental transfusion, were whether breathing occurred during DCC and the length of time after birth before the cord was clamped. In term infants, it appeared that the initial cry was enough to facilitate placental transfusion, as the majority of the decrease in RPV occurred quickly, even if breathing was not sustained. In preterm infants, however, this placental transfusion appeared to take longer, possibly associated with a reduced respiratory effort [19,20]. In addition, the length of DCC may be curtailed because of the desire of attending staff to start early resuscitation, particularly if early respiration is not vigorous. The effect of this shorter duration of DCC combined with irregular respiration on placental transfusion is not clear. Such infants may have been excluded from randomised trials (approximately 20% in the two largest studies) [14,15]. In an observational study of infants < 30 weeks carried out at our hospital, of 46 infants undergoing DCC (40 s or more), 34 established regular breathing during the procedure, whilst 12 (26%) did not. Sixty percent of infants that received DCC and did not breathe received an RBC transfusion compared to 32% in the group that breathed regularly. The median day 1 haemoglobin was significantly higher in this latter group (*p* = 0.003). These differences suggest a larger placental transfusion in the group that breathed during DCC even though the length of DCC was similar [21]. This led us to question whether providing respiratory support to preterm infants who did not establish regular breathing during DCC (but were not otherwise compromised) would allow a greater placental transfusion to take place and subsequently reduce neonatal blood transfusion requirements. We estimated that the assessment and randomisation could be completed within 20 s of birth and planned a further 30 s of DCC, with 1:1 randomisation to either receive respiratory support or no respiratory support during the last 30 s. All study infants would receive a total of 50 s DCC. In PICOT format, this was as follows:

**P** preterm infants < 31 weeks undergoing DCC who did not establish regular respiration by 15 s were randomised to the 

**I** intervention of the T piece intermittent positive pressure ventilation (IPPV) with 30% oxygen compared to the 

**C** control group who received standard care during DCC without IPPV.

**O** outcome assessment was blood transfusion.

**T** time during the neonatal admission.

We anticipated several problems with the study implementation:

*Ergonomics*: Birth, and particularly very preterm birth, is a stressful time with space and equipment limitations. Other investigators have used purpose-built resuscitation trolleys, radiant warmers or other surfaces for infant assessment and management [12,22,23]. However, as described in the “Methods”, we used heated towels on the mother’s thighs or placed the infant at the foot of the bed for vaginal births. We had one skilled operator in charge of the infant who stood next to the surgeon, and in our preliminary trials, this pragmatic approach was satisfactory.

*Monitoring*: Ideally, heart rate and pulse oximetry and temperature should be continuously monitored during this short transition. A recent study found dry electrodes to be the quickest tool for monitoring heart rate (median 13 s) followed by ECG (42 s) and pulse oximetry (105 s). The dry gel electrodes for preterm infants are not yet commercially available and the other methods are too slow for this short period [9,24]. Similarly, with only one neonatal attendee, we could not routinely auscultate or palpate the heart rate. This meant we had to rely on the observation of breathing, activity, tone and colour for monitoring.

*Sterility*: Apart from the neonatal attendee who wore sterile theatre attire, the only contact with the sterile field was the sterile plastic wrap, respiratory circuit and T-piece. A local supplier was able to gas sterilise these components for caesarean deliveries.

*Randomisation and time*: The decision to randomise was undertaken at 15 s and intervention was started by 20 s (this is described in detail below). In simulation testing, we could achieve this with opaque sealed numbered envelopes if we used a colour coded card in the envelope. One of the reasons we did not want to prolong the total DCC time beyond 50 s was because of the safety of the mother and difficulties of safely monitoring potentially compromised infants.

*Temperature*: We applied the sterile thermal wrap immediately after delivery but no other form of heating until the infant was placed under the radiant warmer after cord clamping.

*Efficacy of respiratory support*: Studies have shown T-piece IPPV may be ineffective in delivering adequate tidal volumes in preterm infants, possibly because of an air leak or a closed glottis [25,26]. To help assess respiration, we inserted a colorimetric carbon dioxide detector into the circuit.

*Outcome/sample size*: We calculated a sample size as described in the Methods section. This was based on our previous observational study. Our neonatal blood transfusion guidelines are restrictive and as the day-to-day attending staff were blinded as to whether the infant received the intervention or not, randomisation was expected to reduce bias in deciding whether blood transfusion was indicated.

*Recruitment*: We surmised that either antenatal or deferred consent were required to achieve a realistic study population. Previous studies in our hospital indicated that restricting the study to those with antenatal consent was likely to mean that >50% of potential cases were ineligible.

## 2. ABC Study Protocol

The Assisted Breathing before Cord Clamping study (The ABC Study) (http://www.anzctr.org.au/ACTRN12615001026516.aspx, accessed on 28 January 2020) protocol is described below in more detail. The study design was a single centre randomised controlled trial at a New Zealand centre that had first implemented DCC in 2010. On average 70 < 31 weeks infants are admitted per year, and it was estimated that 50% of infants undergoing DCC would not be breathing regularly by 15 s [13].

### 2.1. Eligibility

Inclusion criteria: Infants < 31 weeks gestation delivered vaginally or by caesarean section and undergoing DCC are eligible once their breathing has been assessed at 15 s of age and the infant is deemed not to have regular rhythmic breathing (chest wall movement).

### 2.2. Exclusion Criteria

- Obstetric reasons: Declined antenatal consent, severe antenatal intrauterine growth restriction (estimated foetal weight < 10th customised centile), twin-to-twin transfusion syndrome, maternal compromise, abruption, en caul, short cord, known severe congenital abnormality.
- Infant reasons: Established regular breathing by 15 s, compromised and in need of immediate resuscitation (apnoeic, flaccid, pale).

### 2.3. Interventions

**Figure 1:**
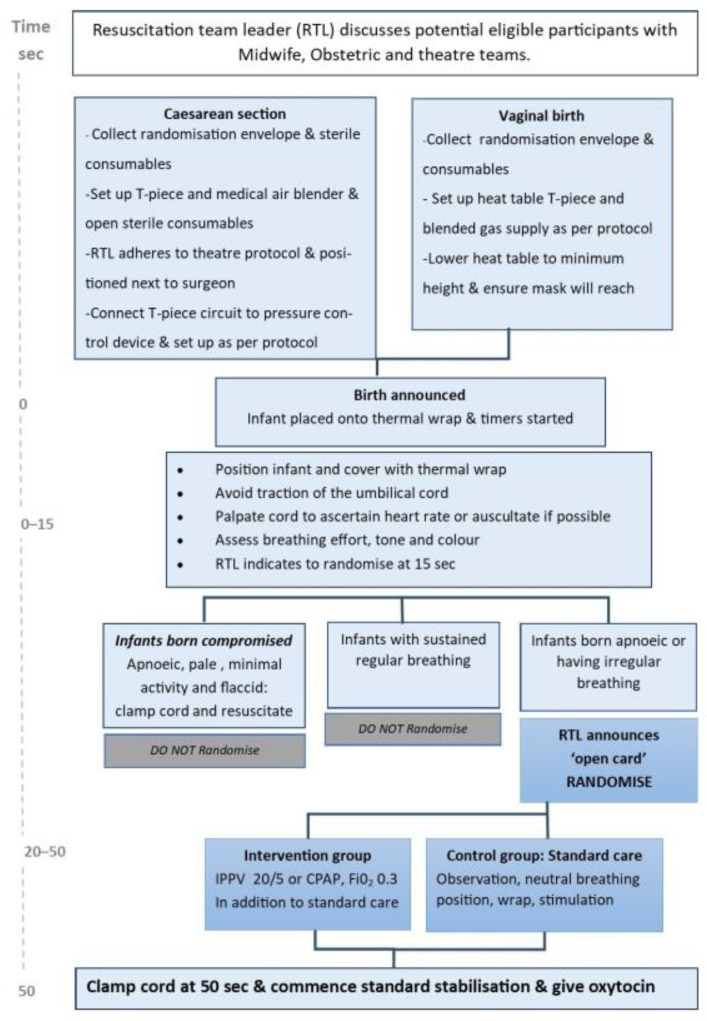
ABC Study flow diagram.

Infants that receive DCC are eligible for randomisation at 15 s of age if breathing (chest wall movement) appears irregular or absent, but the infant is not otherwise compromised (see below). A control group receiving standard treatment will be compared to an intervention arm. Standard treatment consists of the infant being placed supine with the head in a neutral breathing position, placed in a thermal wrap whilst receiving gentle stimulation (face dried, touching or rubbing the torso or back) and completing 50 s DCC. Infants randomised to the intervention arm will receive breathing support in the form of intermittent positive pressure ventilation (IPPV) and continuous positive airway pressure (CPAP) delivered by appropriate mask and T-Piece in addition to standard treatment as described above (Figure 1).

The designated resuscitation team leader (RTL) will collect a sealed, numbered, stratified randomisation envelope for eligible infants < 31 weeks and discuss the study with the midwife and obstetric team antenatally or before delivery.

### 2.4. Equipment

For vaginal deliveries, the T-piece pressure-controlled device (Neopuff, Fisher & Paykel, Auckland, New Zealand) attached to the heat table (Giraffe Warmer, GE Healthcare, Auckland, New Zealand) will be used; the heat table will be lowered to minimum height, next to that of the delivery bed, to allow the circuit and T-piece to reach the foot of the maternal bed. A colorimetric device (Pedicap, Covidien^TM^, Mansfield, MA, USA) will be inserted into the T-piece circuit. A sterile thermal body wrap will be used (Fisher & Paykel, Auckland, New Zealand). Time keeping will be done using the Apgar score timer.

Caesarean section deliveries require sterile consumables, i.e., sterile mask and T-piece circuit, sterile thermal wrap and a colorimetric device. The T-piece and gas blender (without humidity) are attached to a mobile pendant located in the obstetric theatre and checked. The RTL will adhere to surgical protocols.

The T-piece settings include gas flow 10 L/min, set pressure 20/5 cm water and FiO_2_ 0.3. The colorimetric device will be placed between the T-piece and mask.

### 2.5. Procedure

Time keeping: Timers will be started when “baby out” is announced. The research assistant will call out “15 s” and the RTL will respond by announcing “open/randomise” or indicate not to open the randomisation card once the breathing has been assessed. The research assistant will announce when 50 s has elapsed to clamp the cord.

Randomisation will take place after applying thermal wrap, placing infant supine in a neutral position (see above) and assessing breathing as follows:

- Apnoeic infants or those that do not have sustained respiratory effort but no pallor and appear well perfused with reasonable tone are eligible.
- Infants born compromised (apneic, pale, flaccid) and deemed in need of immediate resuscitation are not eligible and have the cord clamped and standard resuscitation commenced [27].

Infants who appear vigorous, with sustained breathing (chest wall movement) at 15 s of age are not eligible for randomisation and neither are infants who initially establish respiration and then became apnoeic after 15 s.

Standard treatment is indicated by a white laminated card whilst the intervention arm is indicated by a blue laminated card (intervention as previously described). During the 50 s DCC, the procedure may be terminated for safety concerns (abruption, clinical deterioration of the mother or infant). After the cord is cut, the infant is placed into a sterile cot (caesarean section) and transferred to a heat table; after vaginal births, the infant will be placed directly onto the heat table. The attending RTL performs the procedure for multiples and a second attendant continues cares after the completion of cord clamping. Oxytocin is to be given after the completion of 50 s DCC for all deliveries as per standard obstetric practice. Standard delivery room management includes humidified Hudson bubble CPAP (6–8 cm; Fisher & Paykel, Auckland, New Zealand) skin servo temperature control, heart rate and saturation monitoring.

### 2.6. Outcome Measures

The primary outcome is red blood cell transfusion rates during the neonatal admission. The proportion of infants transfused as well as the number of transfusions and volume of blood transfused will be compared, with the primary comparison being the proportion transfused in either group. The study centre transfusion guideline will remain unchanged during the planned study period (Table A1 in Appendix A). Infants who receive a transfusion at a surgical centre will be recorded separately to avoid the problem of differing blood transfusion guidelines.

### 2.7. Secondary Outcomes

- Apgar scores and delivery room resuscitation including the requirement for endotracheal intubation.
- Core axillary temperature (digital electronic thermometer) at 5 min of age and on admission to NICU.
- Respiratory support including surfactant, length and type of support.
- Transitional circulation assessed based on blood pressure recorded from arterial lines where possible or peripheral recordings if no arterial lines, acid base, lactate, echocardiogram performed within first 6–12 h if possible, inotropes received (first 48 h) and patent ductus arteriosus treatment rates during neonatal admission.
- Phototherapy received (requirement based on local guidelines) haemoglobin levels on days 1 at 24 h of age and days 2, 3 and 7 (from capillary or venous FBC as preference or capillary gas).
- Neonatal outcomes as defined by local neonatal network coding criteria [28]. These include: chronic lung disease (CLD) defined as respiratory support or oxygen received at 36 weeks corrected gestational age, discharge home with oxygen, intraventricular haemorrhage (IVH) rates (grades III and IV and any grade reported) within first 24 h if possible, day 5 and day 28 cranial ultrasound, necrotising enterocolitis (NEC) defined by modified Bells stage 2 or higher, retinopathy of prematurity (ROP) highest stage recorded and requiring laser therapy, early onset sepsis (EOS) < 48 h of age and late onset sepsis (LOS) defined by positive blood culture or cerebrospinal fluid after 48 h, death during neonatal admission and length of hospital stay in days [28].
- Neurodevelopmental outcome at 2 years of age (for infants ≤ 29 week gestation).

### 2.8. Sample Size

Data from an observational study indicated that 60% of infants that received DCC and did not breathe received a blood transfusion. This compared to 30% transfused in the group that breathed during DCC. Aiming for a similar 50% relative reduction in the proportion transfused would require 100 infants whose primary outcome could be assessed. To allow for cases where outcome assessment may not be possible (deaths, surgical transfer), 120 infants are to be randomised (60 per group). (A total of 100 would give the study 85% power at a two-sided significance level of 0.05). For a secondary composite outcome of death, CLD or severe IVH, the sample size would allow a 36% difference in outcome to be detected (80% power, significance 0.05), assuming the percentage of infants with a composite endpoint will be 58% (based on historic data).

### 2.9. Intervention Assignment

Sequence generation: One-to-one allocation to study groups based on computer generated randomisation sequences with random block sizes. Groups will be stratified into ≤26, >26 and <28 weeks and ≥28 to <31 weeks gestation.

### 2.10. Concealment Mechanism

Randomisation will occur via sequentially numbered brown opaque sealed envelopes containing laminated colour cards to reveal the intervention. The colour code cannot be determined without opening the envelope. Multiples will be randomised as individual participants.

### 2.11. Implementation

The envelope will only be opened once the resuscitation team leader instructs the research assistant to do so. The infants study number will be documented on the birth record and delivery data collection sheet.

### 2.12. Masking

The laminated coloured card and completed randomisation data sheet will be placed in a secure box located in a locked office (only accessible to the research nurse collecting and entering the blinded data into a secure data base). Outcome assessment will be carried out by members of the study team without knowledge of the intervention group.

### 2.13. Data Management

Standardised data collection forms are entered into Research Electronic Data Capture (REDCap, Vanderbilt University Medical Centre, Institute for Clinical and Translational Research, hosted by University of Auckland, Auckland, New Zealand) by an administrator [29].

A data safety monitoring committee (DSMC) was established.

### 2.14. Statistics

Initial chi-square tests for categorical data. Numeric data are to be analysed with Mann–Whitney non-parametric statistics or t test if normally distributed. Secondary analysis is planned using logistic regression (IBM SPSS Statistics 26, IBM Corporation 2020, New York, NY, USA). Two-sided probability (*p* value) < 0.05 will be used to indicate statistical significance.

### 2.15. Ethics

Written informed consent will be obtained before labour where possible. In cases where active labour is in progress; and consent before birth is not possible, approval was obtained from the Northern A Health and Disability Ethics Committee, New Zealand (15/NTA/146AM01) as well as the following local groups to allow randomisation at birth, and obtain postnatal consent for the collection of outcome data: Māori Health Ethics committee, Patient Whanau Centred Care Consumer Council, Maternity consumer group, organisational legal opinion, parental input from Neonatal Unit parents of preterm infants as well as Neonatal, Midwifery, Obstetric and Anaesthetic department stakeholders. In cases where antenatal consent could not be obtained and parents decline consent, the initial data capture sheet will be discarded, and no further data collected.

## Appendix A

**Table A1:** Hospital blood transfusion guideline.

Haemoglobin (Hb) (Haematocrit)	Consider Transfusion
Hb < 70 g/L (Haematocrit ≤ 20%)	Symptomatic anaemia: any of the following without obvious cause: Significant apnoea and bradycardia while receiving caffeine (>9 episodes in 12 h or 2 episodes in 24 h requiring bag mask ventilation). Heart rate > 170 beats per minute or respiration rate > 70 breaths per minute persists for 24 h. Poor weight gain (< 10g/day over 7 days). Increasing ductal shunt and cardiac decompensation. Any positive pressure support. Asymptomatic with reticulocytes < 100,000 × 10^9^/L.
Hb 70–80 g/L (Haematocrit 21–25%)	Symptomatic anaemia as above. CPAP with FiO_2_ 0.25–0.4. Undergoing surgery.
Hb 81–100 g/L (Haematocrit ≤ 25–30%)	CPAP with FiO_2_ > 0.4. Mechanical ventilation with MAP ≥ 7–10 cm H_2_O.
Hb 101–110 g/L (Haematocrit ≤ 21–25%)	Critically unwell with one or more of the following: CPAP and FiO_2_ > 0.4. Ventilation MAP > 10 cm H_2_O. Acute bleeding.
